# Radiation techniques for acromegaly

**DOI:** 10.1186/1748-717X-6-167

**Published:** 2011-12-02

**Authors:** Giuseppe Minniti, Claudia Scaringi, Riccardo Maurizi Enrici

**Affiliations:** 1 Department of Neuroscience, Neuromed Institute, Pozzilli (IS), Italy; 2 Department of Radiation Oncology, Sant' Andrea Hospital, University Sapienza, Rome, Italy

**Keywords:** acromegaly, fractionated stereotactic radiotherapy, radiosurgery, toxicity, GH-secreting pituitary tumors

## Abstract

Radiotherapy (RT) remains an effective treatment in patients with acromegaly refractory to medical and/or surgical interventions, with durable tumor control and biochemical remission; however, there are still concerns about delayed biochemical effect and potential late toxicity of radiation treatment, especially high rates of hypopituitarism. Stereotactic radiotherapy has been developed as a more accurate technique of irradiation with more precise tumour localization and consequently a reduction in the volume of normal tissue, particularly the brain, irradiated to high radiation doses. Radiation can be delivered in a single fraction by stereotactic radiosurgery (SRS) or as fractionated stereotactic radiotherapy (FSRT) in which smaller doses are delivered over 5-6 weeks in 25-30 treatments. A review of the recent literature suggests that pituitary irradiation is an effective treatment for acromegaly. Stereotactic techniques for GH-secreting pituitary tumors are discussed with the aim to define the efficacy and potential adverse effects of each of these techniques.

## Introduction

Acromegaly is a disorder caused by a pituitary GH-secreting adenoma and characterized by high circulating levels of GH and IGF-I. It is associated with increased morbidity and mortality rates, especially due to respiratory, cardiovascular disease, and malignant diseases [1]. Surgery, medical therapy, and radiotherapy (RT) are the available treatments employed with the aim of normalizing GH and IGF-I hypersecretion, controlling pituitary tumor mass effects, preventing recurrences, and improving morbidity. Transsphenoidal surgery is the procedure of choice for the initial management of acromegaly, leading to the remission of disease in 42-65% of patients [2], and achieving a rapid improvement of metabolic and cardiovascular abnormalities [3,4]. Medical therapy, mainly with long-acting somatostatin analogs, permits a normalization of GH/IGF-I hypersecretion in up to 70% of cases with an apparently low incidence of side effects [5]. RT is currently proposed to a subset of patients with persistent active disease after surgery and/or during medical therapy. In most of published studies conventional RT achieves tumor growth control in 85-95% of cases, dropping out GH/IGF-I levels to less than 5 ng/ml in up to 80% of patients 10-15 years after RT [6]. Despite its efficacy, there are concerns about the necessity and potential toxicity of RT and its use remains matter of debate.

More recently, stereotactic radiation techniques have been employed in patients with acromegaly with the aim of treating less normal brain and of minimizing the long-term consequences of RT while improving its effectiveness [7]. Stereotactic radiotherapy can be given as a single treatment (stereotactic radiosurgery-SRS) using either cobalt-60 gamma radiation-emitting sources (Gamma-Knife) or linear accelerator (LINAC), or as fractionated stereotactic radiotherapy (FSRT). Although stereotactic techniques have the clear advantage to offer a more precise radiation delivery compared with conventional RT, the question regarding their superiority and efficacy in the management of patients with acromegaly remains to be demonstrated.

In this review, we present a critical analysis of the more recent available literature in the management of patients with acromegaly, in an attempt to define reasonably objective and comparative information on the safety and efficacy of the individual techniques.

## Fractionated Radiotherapy

Modern RT even without the recourse to stereotactic techniques has seen advances in all aspects of treatment with better immobilization, imaging, planning and treatment. Patients are typically immobilized in a custom made plastic mask with movement limited to 2-5 mm. Tumour localization, initially based on plain X-ray visualization of the pituitary fossa, has improved with the routine use of fused CT/MRI imaging. A margin of 3-10 mm beyond the visible extent of tumour is included in the treatment planning to allow for patient movement and set-up variation during the treatment. Three-dimensional (3D) treatment planning provides more accurate visualization of dose distribution as compared with 2D planning, with the option of giving a more homogeneous dose within the target and lower dose to the organs at risk of radiation toxicity. More precise delivery is achieved conforming the radiation beams to the shape of tumor (conformal radiotherapy) and increasing the number of beams. This results either in reduction of volume of normal brain receiving high dose of radiation or in a greater dose differential between the target and normal brain tissue. The total dose of 45-55 Gy is achieved by daily doses of 1.8-2.0 Gy, with treatment lasting for 5-6 weeks.

Published series assessing the long term effectiveness of conventional RT in patients with acromegaly report tumor control and normalization of GH/IGF-I levels in the region of 80-90% and 50-60% at 10 years, respectively. The reported results are summarized in Table 1[8-15]. Differing from earlier series based on basal GH levels < 5-10 μg/liter to evaluate the biochemical remission of acromegaly after pituitary irradiation, more stringent criteria for disease control are currently used [16].

**Table 1:** Summary of results of recent series on fractionated radiotherapy for GH-secreting pituitary adenoma

Authors	type of	patients	total dose	follow-up	tumor control	biochemical remission	late toxicity (%)	
	RT		(Gy)	median (months)	%	%	visual	hypopituitarism
Barkan et al., 1997 [8]	CRT	38	46	80	NA	5	NA	NA
Thalassinos et al., 1998 [9]	CRT	46	45-50	86	100	25 and 21 at 5 and 10 years	0	30 at 10 years
Barrande et al., 2000 [10]	CRT	128	52	137	NA	35 and 53 at 5 and 10 years	3	50 at 10 years
Biermasz et al., 2000 [11]	CRT	36	40	130	NA	40 and 61 at 5 and 10 years	0	29 and 54 at 5 and 10 years
Cozzi et al., 2001 [12]	CRT	49	45	168	96	10 at 10 years	4	12
Epaminonda et al., 2001 [13]	CRT	67	40-75	120	NA	65 at 15 years	0	NA
Jenkins et al., 2006 [15]	CRT	656	45	84	NA	36 and 64 at 5 and 10 years	0	58 at 10 years°
						74 at 15 years		
Minniti et al., 2005 [14]	CRT	45	45	144	95	29 and 52 at 5 and 10 years	0	45 at 10 years
						77 at 15 years		
Milker-Zabel et al.,2004 [26]	FSRT	20	52.2	61	100	55	5	15
Colin et al., 2005 [27]	FSRT	31*	50.4	80	99	20 and 50 at 5 and 10 years	0	37
Minniti et al., 2006 [28]	FSRT	18*	45	39	98	50 at 5 years*	0	22
Roug et al., 2010 [29]	FSRT	34	54	45	91	30	NA	29

CRT, conventional radiotherapy; FSRT, fractionated stereotactic radiotherapy.

*acromegalic patients included in series of FSRT for either secreting or non secreting pituitary tumors.

°hypogonadism 58%, hypothyroidism 27%, and hyposurrenalism 15%, respectively.

In a series of 45 patients with active acromegaly treated with external beam RT at University of Rome La Sapienza between 1982 and 1994 survival rates were 98%, 95%, and 93%, and local tumor control rates 95% at 5, 10 and 15 years after treatment [14]. Biochemical remission of disease as defined by GH levels below 1 ng/ml during an oral glucose tolerance test (OGTT) was seen in 9% of patients at 2 years, 29% at 5 years, 52% at 10 years, and 77% at 15 years, respectively. IGF-I levels were normal in 8% of patients 2 years after RT, and this proportion increased to 23%, 42% and 61% after 5,10 and 15 years, respectively. In a large retrospective series of 656 patients with acromegaly treated with conventional pituitary irradiation in the United Kingdom the proportion of patients who achieved a safe GH (< 2.5 ng/ml) was 22% at 2 years, 36% at 5 years, 60% at 10 years, and 74% at 15 years [15]. The biochemical remission rates were 35%, 49%, 73%, and 88% at 2, 5, 10, and 15 years for patients with a preirradiation GH levels less than 10 ng/ml, compared with 11%, 27%, 51%, and 69% for patients with preirradiation GH levels of 10-30 ng/ml. Normalization of IGF-I was observed in 38% of patients at 2 years, 50% at 5 years, 63% at 10 years, and 56% at 15 years, respectively. Similar effects of pituitary irradiation on GH and IGF-I levels have been reported in some other retrospective series [10,11].

Analysis of rate of declining of individual GH levels shows that plasma GH declines gradually to approximately 50% of the preirradiation value at 2 years, to 20% at 5 years, and to 10% at 10 years, with a slower decline of IGF-I concentration in the range of 50%-60% at 10 years [10,11,14]. This means that the interval to achieve biochemical remission of acromegaly mainly depends on GH/IGF-I preirradiation levels. Although RT was found effective in the majority of treated patients in most series, few studies reported a less favourable outcome [8,9,12]. Differences in the length of follow-up, disease activity, and biochemical testing procedures may, at least in part, be responsible for these discrepancies.

The risk of late normal central nervous system toxicity of external beam RT to doses less than 50 Gy at 2 Gy per fraction is low, with a reported incidence of optic neuropathy resulting in visual deficits of 1-5%, and a risk of necrosis of normal brain structures of 0-2%. Hypopituitarism represents the most commonly reported late complication of RT, and its frequency increases with longer follow-up, occurring in up to 60% of irradiated patients 10 years after treatment (Table 1). An increased incidence of cerebrovascular accidents (CVA) and related mortality has been reported in patients with acromegaly treated with conventional RT [17,18]. Brada et al [17] found an increased mortality in a series of 334 irradiated patients with a 1.6-fold excess of CVA, and similar results have been reported by others [18]. Since other possible risk factors include GH/IGF-/excesses, hypopituitarism, and extensive surgery, a direct link between RT and cerebrovascular events remains to be proven. Conventional radiation of pituitary tumors has been associated with the development of secondary radiation-induced neoplasm, usually a glioma or a meningioma [19,20]. In a cohort of 426 patients with pituitary adenomas [20] who received CRT at the Royal Marsden Hospital (RMH) between 1962 and 1994, the cumulative risk of second brain tumours was 2.0% (95% CI: 0.9-4.4%) at 10 years and 2.4% (95% CI: 1.2-5.0%) at 20 years. The results are in consistent with the reported cumulative risk of secondary glioma after radiation of 2.7% at 15 years in a cohort of 305 patients with pituitary adenomas [19]. Developmental problems leading to neurocognitive impairment, particularly in children, is a recognized consequence of large volume cranial irradiation [21]; however, there is little evidence that fractionated irradiation for pituitary adenomas may significantly alters cognitive function [22-24].

FSRT is a refinement of high conformal RT with further improvement in immobilization and delivery. Patients undergoing FSRT are usually immobilized in a highly precision frameless stereotactic mask fixation system with a reported accuracy of 1-2 mm [25], so that it is possible to administrate stereotactic irradiation in a number of small doses/fractions. Thus, the principal aim of FSRT is to deliver more localized irradiation as compared with conventional RT, leading to a reduction of the volume of normal brain tissue irradiated to high radiation doses, possibly minimizing the long-term consequences of treatment.

Only few series report on the use of FSRT in patients with GH-secreting pituitary adenomas showing tumor control and biochemical remission rates of 90-100% and 8-55% at a variable follow-up of 30-60 months [26-29] (Table 1). In a series of 18 patients with acromegaly treated with FSRT at Royal Marsden Hospital biochemical remission was achieved in 35% after a median follow-up of 39 months [28]. Actuarial normalization of GH/IGF-I levels was 20% at 3 years and 50% at 5 years. Milker-Zabel et al. [26] reported 5-year local and hormonal control rates of 100% and 80%, respectively, in 20 patients with acromegaly. At a median follow-up of 30 months Roug et al. [29] observed biochemical remission of disease, as defined by suppressed GH at OGTT and normal IGF-I levels adjusted for age, in 30% of 34 patients with active acromegaly, being 24%, 38% and 64% after 1,3 and 5 years, respectively. An additional 20% of patients achieved normal GH and IGF-I levels with the use of somatostatin analogs during the follow-up.

A low radiation-induced toxicity has been reported after FSRT. Hypopituitarism is the most common complication of treatment and has been reported in 15-37% of patients at median follow-up ranging from 39 to 80 months, whereas the reported incidence of optic neuropathy is 1-5%. The incidence of hypopituitarism is likely to remain the major late effect of FSRT since it does not result in a significant reduction of dose to the hypothalamus and the residual normal pituitary tissue. Although no cases of CVA and second tumors have been reported after FSRT, the incidence of the former increases with time, and secondary tumors usually occur with many years delay. Although treating less normal brain at high radiation doses may translate in a reduction of the development of such radiation induced complications, large series and longer follow-ups need to demonstrate these potential clinical advantages. Similarly, the lack of formal cognitive function testing and quality of life assessment in all published series does not allow for definitive conclusion about the potential superiority of stereotactic techniques as compared with 3D conformal RT, and this will need to be addressed in future studies.

Intensity-modulated radiation therapy (IMRT) represents an advanced form of 3D conformal RT with the potential to achieve a much higher degree of target conformity while minimizing radiation exposure to surrounding normal tissues, especially for tumors with complex shapes and concave regions close to sensitive structures. IMRT uses a series of multiple subfields created by a multileaf collimator (MLC) which move under computer control creating modulated fields. IMRT treatment plans are generated using inverse planning system, which uses computer optimization techniques to modulate intensities across the target volume and sensitive normal structures, starting from a specified dose distribution. IMRT may result in a more conformal and better coverage than 3D conformal RT and therefore is able to spare more normal brain. In 34 patients with pituitary adenoma treated with IMRT at a median follow-up of 42 months local control was 89% [30]. However, there are no reported clinical data on IMRT in acromegaly, and currently, it is not possible to conclude that IMRT confers any advantage over other techniques with respect to either hormonal control or toxicity.

Particle radiation has been also applied successfully in the treatment of pituitary adenomas. The physical properties of proton irradiation can offer superior conformality in dose distribution when compared to 3D conformal RT and IMRT. Distribution of low and intermediate doses to portions of the brain in children irradiated for common brain tumors are significantly lower with protons when compared with photons [31], and the advantage becomes more apparent for large volumes. Proton therapy can be delivered as SRS or as FSRT with the same immobilization systems and target accuracy of photon techniques.

Petit JH et al [32] reported on 22 patients with persistent acromegaly who were treated with single fraction proton radiosurgery at Massachusetts General Hospital. Using a median dose of 20 GyE biochemical remission was achieved in 50% of patients, with a median time to complete response of 30.5 months. One-third of patients developed at least one new pituitary deficiency, requiring medical therapy. In a small series of 11 acromegalic patients treated with fractionated proton beam irradiation at a median time of 83 months hormonal normalization occurred in 45% of patients, with an actuarial rate of 23% at 5 years [33]. Currently, no data suggest the superiority of protons in the treatment of pituitary tumors as compared with other radiation techniques.

## Stereotactic radiosurgery (SRS)

SRS is given using either a multiple cobalt-60 (^60^Co) gamma radiation-emitting sources gamma knife (GK) or a modified linear accelerator (LINAC). GK is the most widely published radiosurgical methodology used to treat pituitary adenomas. In its most common design, a total of 201 sources of ^60^Co are arranged in a hemisphere and focused with a collimator helmet on a single or multiple fixed points (isocenters). CT localization and computerized 3D planning are used to determine the optimal number and distribution of isocenters, and this can be aided by selective occlusion of collimator apertures. During SRS, patients are usually immobilized in a fixed frame with a positioning accuracy of < 1 mm. Similar dose distribution can be obtained with a LINAC using multiple noncoplanar arcs of rotation or multiple noncoplanar fixed beams.

SRS has been extensively employed in the last two decades in patients with residual pituitary tumors. At a median follow-up ranging between 31 and 60 months the reported tumor growth control following SRS in patients with acromegaly is between 88 and 97% [34-48] (Table 2). A variable reduction in tumor size has been observed in 30-60% of patients after the treatment.

**Table 2:** Summary of results of recent series on stereotactic radiosurgery for GH-secreting pituitary adenomas

Authors	patients	type of	total dose	follow-up	tumor	biochemical remission	late toxicity (%)	
		SRS	(Gy)	median (months)	control (%)	(%)	visual	hypopituitarism
Attanasio et al., 2003 [34]	30	GK SRS	20	46	100	30 at 5 years	0	6.7
Jane et al., 2003 [35]	64	GK SRS	15	> 18	NA	36	0	28
Castinetti et al., 2005 [36]	82	GK SRS	26	49.5*	NA	17	1.2	17
Gutt et al., 2005 [37]	44	GK SRS	23	22	100	48	NA	NA
Kobayashi et al., 2005 [38]	67	GK SRS	18,9	63	100	17	11	15
Jezkova et al., 2006 [39]	96	GK SRS	32	53.7	100	44 at 5 years	0	27.1
Voges et al., 2006 [40]	64	LINAC SRS	16,5	54.3	97	14 and 33 at 3 and 5 years	1.4	13 and 18 at 3 and 5 years
Petit et al., 2007 [32]	22	PSRS	20 GyE	75.6	100	59	0	38
Pollock et al., 2007 [41]	46	GK SRS	20	63	100	11 and 60 at 2 and 5 years	0	33 at 5 years
Vik-Mo et al., 2007 [42]	53	GK SRS	26.5	67	100	58 and 86 at 5 and 10 years	3.8	10 at 5 years
Jagannathan et al., 2008 [43]	95	GK SRS	22	57	98	53	4	34
Losa et al., 2008 [44]	83	GK SRS	21,5	69	97	52 and 85 at 5 and 10 years	0	10 at 10 years
Ronchi et al., 2009 [45]	35	GK SRS	20	114	100	46 at 10 years	0	50
Wan et al., 2009 [46]	103	GK SRS	21,4	67	95	37	0	6
Hayashi et al., 2010 [47]	25	GK SRS	25.2	36	100	40	0	0
Iwai et al., 2010 [48]	26	GK SRS	20	84	96	17 and 47 at 5 and 10 years	0	8

*mean follow-up; NA not assessed.

SRS, stereotactic radiosurgery; GKS, Gamma Knife radiosurgery.

PSRS; proton stereotactic radiosurgery.

Biochemical remission of disease has been reported in 35-100% of patients with GH-secreting adenomas. The variable rate of control disease may reflect the different lengths of follow-up and criteria used to define the biochemical control of disease, making difficult the evaluation of the real efficacy of SRS. Nevertheless, when stringent criteria of cure as defined by suppressed GH levels during OGTT and normal age-corrected IGF-I levels are considered, the 5-year actuarial biochemical remission has been reported in 30-60% of patients following SRS, including patients who achieved normal GH/IGF-I levels during medical treatment with somatostatin analogs, and normalization of GH/IGF-I levels continues throughout the follow-up period [36,40-42,44] (Table 2).

Losa et al [44] in a retrospective analysis of 83 patients with acromegaly treated with GK SRS at University of Milan San Raffaele between 1994 and 2006 have reported actuarial biochemical remission rates of 30%, 52% and 85% at 3, 5 and 10 years, respectively. Jagannathan et al [43] observed normalization of the serum IGF-1 in 53% of 95 patients treated with GK SRS and at least 18 months of follow-up. The mean time to remission was 30 months; twelve patients achieved endocrine remission within the first year of treatment, 28 within 2 years, and 34 within 3 years, respectively. Jezkova et al [39] in a series of 96 patients reported hormonal remission rates of 45% at 3 years, 58% at 5 years, and 57% at 8 years, respectively. The median time to achieve GH suppression < 1 μg/l during an OGTT and normal IGF-I was 66 months. Similar biochemical remission rates in the range of 45-60% at 5 years have been shown by others [41,42], although lower rates have been reported in some series [34,36,40,48]. There are only few studies on the efficacy of LINAC SRS for the treatment of GH-secreting pituitary adenomas [40]; in general, they show comparable efficacy to GK SRS.

Several factors including preirradiation GH/IGF-I levels, the use of somatostatin analogs, and radiosurgical dose have been correlated with the endocrinological outcome after SRS, although disagreement exists across the published series.

High GH and/or IGF-I levels have been found independently associated with worse SRS outcome in some series [36,39,41,44], similar to that reported after conventional RT [10,11,14,15]. Losa et al [44] reported a median time for remission of 37 months for patients with pre-treatment GH levels ≤ 7 μg/liter as compared with 93 months for patients with GH levels > 7 μg/liter. IGF-I levels ≤ 1.8 times the upper limit of normal reached remission at a median time of 36 months as compared with 90 months for patients with > 1.8 times the upper limit of normal. Similarly, in a retrospective analysis of 46 consecutive patients treated by SRS between 1991 and 2004 at Mayo Clinic, preirradiation IGF-I levels were independently correlated with biochemical remission. The 3-year and 5 year biochemical remission rates were 40% and 90% for patients with IGF-I levels less than 2.25 times the upper limit of normal, and 23% and 38% with IGF-I levels greater than 2.25 times the upper limit of normal, respectively. Although no relationship between baseline hormonal levels and remission of acromegaly has been reported in few series [34,42], it seems reasonable that patients with near-normal GH and IGF-I levels are more likely to achieve hormonal remission than patients with markedly abnormal pretreatment levels.

Whether the concomitant use of somatostatin analogs at the time of SRS is a negative predictor of endocrine normalization remains matter of debate. In Landolt at al. [49] and Pollock et al. [41] series the use of suppressive medications at the time of SRS negatively correlated with biochemical remission of disease and increased the time to hormonal normalization. In contrast, other authors failed to show any detrimental effect of medical treatment on outcome [34,36,44]. Although somatostatin analogs withdrawal before SRS has gained an increase acceptance in clinical practice, future prospective studies are needed to elucidate the issue.

A variable dose of 18-32 Gy has been employed for SRS in acromegaly. With some exceptions, marginal dose to the tumor was not independently associated with higher rate of remission or faster normalization of hormone hypersecretion [34-48]. Currently, a marginal dose of about 20-25 Gy seems appropriate to achieve either tumor control or hormonal normalization.

The reported overall rate of serious complications after SRS is low (Table 2). The main complication is hypopituitarism which is reported in 0-47% of patients, with higher rates in those series with longer median follow-up (Table 2). Pollock et al. [41] reported that one third of 39 patients with acromegaly had a new pituitary deficit following GK SRS, with an actuarial incidence of new anterior pituitary deficits of 10% at 2 years and 33% at 5 years, respectively. In a series of 95 patients with acromegaly treated with GK SRS new endocrine deficiencies were observed in 34% of patients. Incidence was only 5% at 12 months after SRS, however increased to more than 1/3 in patients with a follow-up longer than 49 months. A similar incidence of hypopituitarism at 5 years in the region of 20-40% has been observed in few other series [39,42,45], suggesting that it will likely increase significantly over time.

Other treatment-related complications occur rarely after SRS. To minimize visual complications the dose received by optic apparatus is usually restricted to less than 8-10 Gy. In clinical practice this means that a distance between tumor margin and optic apparatus should be at least of 2-3 mm to avoid the risk of visual deterioration while delivering an effective dose of 16-20 Gy to the tumor. Cavernous sinus is frequently irradiated at high dose in patients with residual pituitary tumor, although cranial neuropathies, brain radionecrosis, and carotid artery stenosis have been reported infrequently following SRS. Loeffler et al. [50] reported two cases of secondary brain tumors after SRS for a pituitary adenoma. The risk to develop a new tumor after SRS appears to be significantly less than that seen following fractionated RT [20], however the relatively short length of follow-up of most published series does not allow for any definitive conclusion.

CyberKnife (Accuray, Sunnyvale, CA) is a relatively new technological advancement in radiation therapy in which a miniaturized low energy linear accelerator is mounted on a robotic arm. The main advantage of Cyberknife system is that it allows for frameless image-guided radiation treatments achieving the same level of targeting precision as frame-based SRS. It can be used for multisession SRS (hypofractionated stereotactic radiotherapy) in patients with tumors involving the optic apparatus and who are not suitable for SRS [51]. Initial experiences with the application of CyberKnife SRS or hypofractionated SRT in treating patients with acromegaly are promising [52,53]. In a report of nine patients with acromegaly treated with CyberKnife to doses of 18-24 Gy in one to three fractions, biochemical remission was observed in 4 patients at a mean follow up of 25.4 months [53]. The efficacy of hypofractionated treatment schedules which may offer a reduced risk of radiation-related adverse effects as compared to single fraction radiosurgery needs to be evaluated in future studies.

A comparison of SRS with FSRT in terms of endocrinological outcome and toxicity is difficult to perform since the choice of the different stereotactic treatment modalities is based on different tumor characteristics: patients with large tumors in close proximity of optic apparatus are likely to be treated with FSRT than SRS. In current practice SRS is usually offered to patients with relatively small adenomas less than 3 cm in size and more than 2-3 mm away from the optic apparatus in order to avoid irradiation of the optic apparatus beyond single doses of 8-10 Gy. In contrast, there is no restriction to the size and the position of adenomas suitable for standard dose fractionated RT, since the treatment is delivered within the radiation tolerance limits of neural tissue, including the optic apparatus. Although early series reported a faster decline of serum GH concentration after GK SRS as compared with FSRT [49,54], the superiority of SRS in terms of time to hormonal normalization remains to be demonstrated. Recent series have in fact showed that the rate of decline of GH/IGF-I levels observed following SRS is in the same region of that observed following fractionated RT, suggesting that the variable time to hormonal normalization is more dependent on preirradiation GH/IGF-I levels than differences in radiation techniques [34,36]. A lower incidence of hypopituitarism has been suggested with the use of SRS as compared with FSRT, although this is likely to reflect different patient selection. SRS is usually used to treat patients with smaller tumors than those treated with FSRT. Prospective studies comparing SRS with fractionated stereotactic radiotherapy in patients with pituitary adenomas similar in size would be of value to help define the long-term efficacy and toxicity of the techniques.

## Conclusion

Radiation is highly effective in the management of patients with persistent active acromegaly after surgery and/or during medical therapy. Long-term data clearly indicate that conventional RT is able to achieve biochemical remission of disease in 50-60% of patients after 10 years, with an acceptable incidence of complications. Stereotactic techniques (SRS and FSRT) offer a more localized irradiation compared with conventional RT and have the potential of reducing the risk of long term radiation induced morbidity. Currently, SRS and FSRT represent common treatment modalities of irradiation for GH-secreting pituitary tumors, providing a comparable high rates of tumor control and endocrinological remission with low morbidity. The choice of the radiation technique should be based on tumor characteristics. In most centres SRS represent a convenient treatment for patients with relatively small residual adenomas not in close proximity of the optic chiasm, while FSRT is usually reserved to patients with larger GH-secreting tumors not amenable to SRS. Efficacy and toxicity of hypofractionated treatment schedules need to be explored in future studies.

## Competing interests

The authors declare that they have no competing interests.

## Authors' contributions

GM and CS performed the database search, critically reviewed the existing data and drafted the manuscript. RME critically reviewed/revised the article. All authors read and approved the final manuscript.
